# Agent-specific training directs distinctive immune response against *Listeria monocytogenes* infection

**DOI:** 10.3389/fimmu.2026.1868138

**Published:** 2026-07-24

**Authors:** Marie Kim, Hailey G. Main, Aaron P. Esser-Kahn

**Affiliations:** 1 Pritzker School of Molecular Engineering, University of Chicago, Chicago, IL, United States; 2 Department of Chemistry, University of Chicago, Chicago, IL, United States; 3 CZI Biohub Investigator, Chicago, IL, United States

**Keywords:** flunisolide, infection, innate immune memory, myricetin, trained immunity

## Abstract

The nonspecific protection provided by the innate immune system can be enhanced through training with stimuli, a phenomenon called ‘trained immunity’. However, it remains unknown if distinct training programs with various functional outcomes can be induced. Here, we show that two training agents, flunisolide and myricetin, distinctively train and modulate the immune response against *in vivo Listeria monocytogenes* infection. Training with either agent led to significant reduction in pathogen burden but differed in the resulting immune functions. Training with myricetin led to the expansion of myeloid progenitor lineages, rapid cell recruitment, and enhanced phagocytosis. In contrast, training with flunisolide led to expansion of stem cell and multipotent progenitor populations, increase in antigen processing, and faster T cell expansion. We provide preliminary evidence that this divergent training outcomes may arise from agent-specific modulation of transcription factors. This study importantly shows that training can be directed at both the innate response and the onset of the adaptive response, highlighting the potential of modulating distinct training programs to influence protective immune responses.

## 1 Introduction

Innate immunity has traditionally been regarded as a rapid yet nonspecific form of resistance to pathogens, lacking memory and distinct response profiles. However, recent discoveries indicate a form of innate memory, termed ‘trained immunity’, that is mediated by epigenetic and metabolic reprogramming. This innate immune training can be translated into enhanced inflammatory and antimicrobial responses upon a secondary challenge, leading to broader host protection. Various stimuli have been reported to induce innate training, such as vaccination (1–3), *in vivo* infection (3–8), and pathogen-derived molecules (7, 9–16). The innate training induced by these stimuli resulted in durable, robust cytokine production and effector functions (17–20). However, adverse effects, such as excessive inflammation (21–24) or maladaptive training (25, 26), arise from the inflammatory nature of these stimuli. Currently, there is little understanding of how distinct training agents encode unique training programs. Furthermore, the broadly reported agents of innate training have not identified a functional mechanism to enhance adaptive responses. However, recent studies have demonstrated a pivotal role for the adaptive immune system in shaping BCG-trained innate immunity, underscoring the importance of understanding the feedback interactions between the innate and adaptive immune responses in the context of trained immunity (27, 28).

Studies have shown that macrophages exhibit stimulus-specific responses, mediated by pathway branching (29–31). These results suggest innate training can also lead to unique, agent-specific training outcomes. Recently, our lab has reported on non-pathogen-derived, small-molecule inducers of innate training (32). Unlike classical training agents, these molecules avoid an initial inflammation reaction during the training process, but train cells to produce enhanced levels of pro-inflammatory cytokines upon stimulation. Notably, distinct changes in chromatin accessibility and transcription factor (TF) footprints were observed between two of the identified molecules, flunisolide and myricetin, indicating distinct response categories. Such a differential training outcome was also reported in a recent study using β-glucan and muramyl dipeptide (MDP) (33). The question remains on how the epigenetic modifications induced by these small molecules translate into system-level effects against live pathogen infection.

Here, we demonstrate that training with flunisolide or myricetin induces distinct cellular responses that confer distinct host protection against a subsequent live infection. Using live *Listeria monocytogenes* (Lm) as our infection model, we demonstrate agent-specific effects on multiple aspects of myeloid function, including phagocytosis, cell recruitment, and chemokine production. We also found that these agents alter cellular responses in both the bone marrow (BM) and the infected peripheral tissue. Furthermore, we show that innate immune training can direct responses at the onset of an inflammatory response and subsequent changes in the adaptive response. These distinct training outcomes may be due to agent-specific alteration in the mitogen-activated protein kinase (MAPK) signaling pathway previously implicated in trained immunity (31, 34). Overall, our results provide mechanistic insight into how different training modalities can shape immune responses and offer potential strategies to develop broader prophylactic approaches against pathogenic infections.

## 2 Materials and methods

### 2.1 Mouse training and infection

Female C57BL/6J mice (6 weeks of age) were purchased from the Jackson Laboratory and were allowed to acclimate for 7 days in the vivarium facility before proceeding with experiments. To train mice with flunisolide or myricetin, Captisol^®^ was used to solubilize the small molecules in 0.9% saline. Flunisolide or myricetin was first dissolved in DMSO at a concentration of 50 mg/ml. A separate stock solution of Captisol^®^ was made at 20% w/v using sterile 0.9% saline. Next, 100 µL of the DMSO stock (50 mg/ml) was mixed with 900 µL of 20% w/v Captisol^®^ to create a 5 mg/ml working stock of flunisolide or myricetin. For intranasal administration, 5 mg/ml working stock of flunisolide or myricetin was diluted with 0.9% saline to 0.1 µmol in 10 µL to administer per mouse. For rhodamine labeling, 2 mg of NHS-Rhodamine (ThermoFisher) was dissolved in 1 mL of 20% w/v Captisol^®^ and 10 µL was administered intranasally. For intranasal administration, mice were anesthetized with isoflurane and administered 10 µL of flunisolide or myricetin or infected with 5 x 10^6^ CFU of *Listeria monocytogenes* suspended in 10 µL of sterile 0.9% saline.

### 2.2 Listeria culture

Bacterial strain *Listeria monocytogenes* (EGDe) was purchased from ATCC (BAA-679). Bacteria were cultured in Brain Heart Infusion (BHI) broth (BD) at 37 °C, shaking (225 rpm). For expansion, bacteria were cultured overnight. For infection, bacteria were cultured overnight in 6 mL of BHI broth in a 14 mL round bottom tube, then 1 mL of the overnight culture was added to fresh 100 mL BHI broth in a 500 mL conical flask and cultured for 4 h.

### 2.3 Flow cytometry

Cells were suspended in PBS and stained with Zombie Aqua fixable dye (1:500, BioLegend) for 30 min at room temperature, then washed with flow buffer (PBS^-/-^ + 0.5% BSA + 10mM HEPES). Cells were resuspended in 100 µL of flow buffer and blocked with TruStain FcX antibody (BioLegend) on ice for 10 min, then stained on ice for 15 min with different combinations of antibodies against CD45 (Spark UV 387), CD3 (APC), CD4 (BV711), CD8 (APC/Fire810), CD11c (BV650), F4/80 (BV421), NK1.1 (PerCP/Cy5.5), CD19 (PE/Dazzle594), Ly6G (APC/Cy7), CD11b (BV605), Ly6C (AlexaFluor700), CCL2 (FITC), CCL22/MDC (AlexaFluor594), CXCL9 (PE), CCL5 (PE/Cy7), and MHCII (Spark Red 718) (all from BioLegend) (**Supplementary Figure 4**). For bone marrow profiling, cells were stained with antibodies against Sca-1 (PerCP/Cy5.5), CD34 (APC), CD16b (APC-Cy7), cKit (PE), CD150 (BV421), CD48 (BV605), CD127 (BV711). For lineage markers, cells were primary stained with biotin antibody cocktail against CD3 (clone 145-2C11), Ly6G/Ly6C (clone RB6-8C5), CD11b (clone M1/70), CD45R/B220 (clone RA3-6B2), and TER-119 (clone TER-119), then secondary stained with anti-biotin antibody (FITC) (all from BioLegend). Bone marrow cells were categorized with the following combination of surface markers: LSK (Lin^-^ Sca-1^+^ cKit^+^), HSC (Lin^-^ Sca-1^+^ cKit^+^ CD150^+^ CD48^-^), ST-HSC (Lin^-^ Sca-1^+^ cKit^+^ CD150^-^ CD48^-^), HPC-2 (Lin^-^ Sca-1^+^ cKit^+^ CD150^+^ CD48^+^), MPP (Lin^-^ Sca-1^+^ cKit^+^ CD150^-^ CD48^+^), MP (Lin^-^ Sca-1^-^ cKit^+^), CMP (Lin^-^ Sca-1^-^ cKit^+^ CD34^+^ FcγRIIIb^lo^), GMP (Lin^-^ Sca-1^-^ cKit^+^ CD34^+^ FcγRIIIb^hi^), MEP (Lin^-^ Sca-1^-^ cKit^+^ CD34^-^ FcγRIIIb^lo^), and CLP (Lin^-^ Sca-1^lo^ cKit^lo^ CD127^+^) (**Supplementary Figure 5**). Cells were washed once after staining and resuspended in 300 µL of flow buffer for analysis. For intracellular staining, cells were fixed with 4% PFA (PBS) for 15 min at room temperature and washed with PBS; then, permeabilized with 0.2% (v/v) Tween 20 (PBS) for 10 min at room temperature and washed with PBS, before proceeding with blocking and staining. Cells were analyzed with Aurora 5L (Cytek) spectral flow cytometer at the University of Chicago Cytometry and Antibody Technology Facility. Data was analyzed using FlowJo software (BD Biosciences).

### 2.4 Cell and tissue collection

Blood was collected via cardiac puncture. Bronchoalveolar lavage fluid (BALF) was collected by washing the lungs with 3 mL of PBS. Mice were perfused with ice cold PBS^-/-^ (2 mM EDTA) before lungs were collected in cell media. Blood was centrifuged at 400 x g for 10 min at 4 °C to collect plasma and leukocytes. BALF was centrifuged at 350 x g for 7 min at 4 °C to collect the supernatant and cells. Lungs were minced using scissors and digested in Collagenase D (Roche) for 30 min at 37 °C. Digested lungs were filtered through 70 µm filters and washed three times to achieve single cell suspensions. For magnetic bead isolation, single cell suspensions were suspended in isolation buffer (PBS^-/-^ + 0.5% BSA + 2 mM EDTA) and incubated with F4/80 biotin-antibody cocktail (MojoSort Mouse F4/80 Selection Kit, BioLegend) on ice for 15 min. Cells were washed and resuspended in isolation buffer and incubated with streptavidin nanobeads on ice for 15 min. Then, cells were washed and resuspended in 1 ml of isolation buffer and separated using a magnet for 15 min. Supernatant was removed and the remaining fraction was resuspended in isolation buffer and separated again on a magnet for 15 min. After removing the supernatant, the remaining cells were used for downstream assays.

### 2.5 T cell proliferation assay

Spleens were harvested from wild type mice to get single cell suspensions. Cells were seeded at 250,000 cells per well in a 96-well round bottom culture plate. Media (RPMI + L-glu, 10% FBS, 2-mercaptoethanol, NEAA, antibiotic-antimycotic) was supplemented with 10 ng/mL of recombinant mouse IL-2 (R&D Systems). Small molecules (2 nmol) or vehicle control were added to the wells every other day, for a total of 3 doses. At 7 days after the last training dose, cells were stained with 5 µM of CellTrace dye (ThermoFisher) for 20 min at 37 °C. After washing, were infected with *Listeria monocytogenes* at MOI of 1. After 2 h infection, the cells were treated with gentamicin (50 µg/ml) to remove any extracellular bacteria and were replenished with fresh media. Cells were cultured for 3 additional days after infection then were fixed with 4% PFA (PBS) for 15 min at room temperature before analysis with Aurora 5L (Cytek) spectral flow cytometer.

### 2.6 APC antigen processing assay

Spleens were harvested from wild type mice to get single cell suspension. Cells were seeded at 200,000 cells per well in a 96-well round bottom culture plate. Flunisolide (2 nmol) or vehicle control was added to the wells every other day, for a total of 3 doses. On day 7 after the last training dose, DQ-Ova (1 µg/well, Thermo Fisher) was added to each well and cells were incubated for 4 h before washing with PBS. Cells were stained with a viability dye (Zombie Violet, 1:500, BioLegend) for 30 min at room temperature, washed with PBS, and then fixed with 4% PFA (PBS) for 10 min at room temperature. After washing with PBS, cells were blocked with TruStain FcX antibody (BioLegend) on ice for 10 min, then stained on ice for 15 min with a combination of antibodies against CD11c (Alexa Fluor 647), CD11b (BV605), and F4/80 (BV510) before analysis with Aurora 5L (Cytek) spectral flow cytometer.

### 2.7 Phagocytosis assay

Cells were isolated from infected lungs into single cell suspension in media (RPMI + L-glu, 10% FBS + antibiotic-antimycotic) and mixed with pHrodo Green *S. aureus* BioParticles (ThermoFisher), then incubated at 37 °C for 2 h. After incubation, cells were washed and fixed in 4% PFA, then analyzed with Aurora 5L (Cytek) spectral flow cytometer.

### 2.8 Chemokine measurement

Chemokine levels of CXCL1 (KC), CCL5 (RANTES), CCL20 (MIP-3a), CCL11 (Eotaxin), CCL17 (TARC), CCL2 (MCP-1), CXCL9 (MIG), CXCL10 (IP-10), CCL3 (MIP-1a), CCL4 (MIP-1b), CXCL13 (BLC), CXCL5 (LIX), CCL22 (MDC) were measured with LEGENDplex MU Proinflamm Chemokine Panel 1 per manufacturer’s instructions (BioLegend). Data was analyzed using LegendPlex Data Analysis Software.

### 2.9 BMDM culture and Western blot

Bone marrow was flushed from femurs and tibias of female C57BL/6J mice using 25G needle and L-15 media. Red blood cells were lysed using ACK lysis buffer. Cells were resuspended in growth media (RPMI + 10% FBS + 1% penicillin-streptomycin + 2-mercaptoethanol + glutamine) supplemented with 25 ng/ml M-CSF (BioLegend) to 1 million cells/mL. On day 0 of culture, 10 mL of cell suspension was added to non-TC petri dish (100 mm). Growth media supplemented with M-CSF was changed fresh on day 3 and day 5 of culture. On day 7, cells were removed from the dish by removing media, washing with PBS, then adding 10 mL of ice cold 5 mM EDTA and incubated for 10 min at 4 ˚C. Cells were reseeded into 6-well plates at 1 million cells/well. For immortalized BMDMs (passage 4, gifted from Jonathan Kagan, Harvard University), cells were passaged for a minimum of two times from frozen stock in growth media (DMEM media + 10% HI-FBS + 1% antibiotic-antimycotic). Cells were detached with 2.5 mM EDTA and seeded into 6-well plates at 1 million cells/well. BMDMs or iBMDMs were treated with vehicle control (DMSO) or 10 µM of flunisolide or myricetin for 24 h. After 24 h, cells were washed and stimulated with LPS (100 ng/mL) or vehicle control (PBS). After 1 h of stimulation, cells were washed and harvested with 200 µL of ice cold RIPA buffer (protease inhibitor + PhosSTOP). Protein concentration of cell lysates was quantified using BCA assay. Samples were boiled with 4x Laemmli loading buffer (10% 2-mercaptoethanol) at 95 ˚C for 5 min. A total of 10 µg of protein of each sample or 10 µL of Precision Plus Protein Unstained Standards (Bio-Rad) was loaded into 4-15% Mini-PROTEAN TGX Gels (10 well, Bio-Rad) and separated for 40 min at 150 V then transferred to nitrocellulose membranes. Membranes were blocked with 5% BSA (TBST) for 1 h at RT and incubated with primary antibodies (1:1000 for rabbit monoclonal anti-NF-kappaB p65, anti-phospho-NF-kappaB p65, anti-ATF-2/ATF-7, anti-phospho-ATF-2(Thr71)/ATF-7(Thr53), Cell Signaling Technology) or 1:500 for mouse monoclonal anti-β-actin primary antibody, Sigma Aldrich) overnight (16 h) at 4 ˚C. Membranes were washed and incubated with HRP-conjugated secondary antibodies (1:1000 for anti-rabbit IgG HRP-linked antibody, Cell Signaling Technology or 1:500 for anti-mouse IgG HRP-linked antibody, Cell Signaling Technology) for 1 h at RT. Membranes were washed and developed with SuperSignal West Pico PLUS substrate (Thermo Scientific) for 5 min then imaged. Membranes were stripped using Restore PLUS Stripping Buffer (Thermo Fisher) for 15 min at RT. Image was processed using Fiji software.

### 2.10 Statistical analysis

The data are expressed as the means with the standard error of the means (± SEM). Data was tested for normality using Shapiro-Wilk test and tested for equal variance using F test or Barlett’s test. Statistical significance was determined by using the appropriate test (unpaired t-test, Welch’s t-test, Mann-Whitney test, Ordinary ANOVA with Tukey’s correction, Welch’s ANOVA with Dunnett’s correction, or Kruskal-Wallis test with Dunn’s correction, two-way ANOVA with Tukey’s correction) depending on normality and variance with Prism (GraphPad). Differences were considered statistically significant if P < 0.05.

## 3 Results

### 3.1 Significant reduction of pathogen burden is observed in mice trained with flunisolide or myricetin

**Figure 1:**
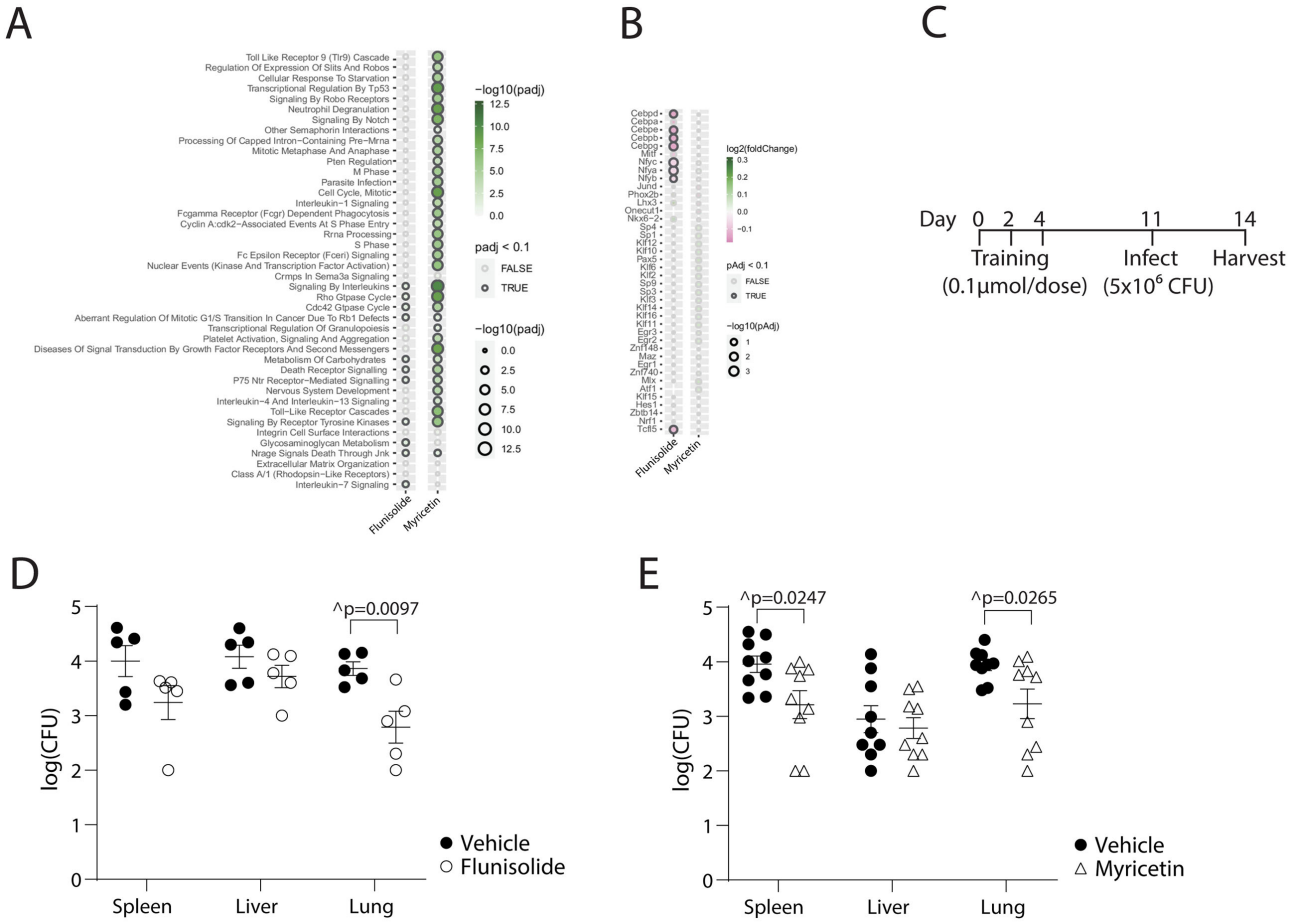
Training with small molecules significantly lowers pathogen burden. Our previous report shows distinct, differentially accessible pathways **(A)** and transcription factor (TF) footprints **(B)** after training with flunisolide or myricetin. **(C)** Experimental design of *in vivo* training and *Listeria monocytogenes* infection. CFU counts from infected spleen, liver, and lung in mice trained with flunisolide **(D)** or myricetin **(E)**. Organs from mice infected with *L. monocytogenes* were harvested on day 3 post-infection. Data is represented as mean ± SEM. ^Unpaired t-test. Panels **A** and **B** we adapted with permission from Knight et al., High-throughput screen identifies non inflammatory small molecule inducers of trained immunity, doi: 10.1073/pnas.2400413121.

Our previous report showed distinct training responses to flunisolide and myricetin, as reflected in chromatin accessibility and TF footprints (**Figures 1A, B**) (32). We observed differential changes in pathways related to inflammatory response, including toll-like receptor (TLR) signaling and phagocytosis. While we measured a significant increase in tumor necrosis factor (TNF)-α and interleukin (IL)-6 production upon lipopolysaccharide (LPS) stimulation in a previous study, we had yet to assess the extent of this training outcome. We hypothesized that the distinct epigenetic changes induced by flunisolide and myricetin would translate into distinct cellular changes in the host, thereby enhancing protection against a live pathogen infection.

Before we could test this hypothesis, we needed a carrier system to administer these agents *in vivo*, due to the hydrophobicity of flunisolide and myricetin. To accurately load and dose the two compounds at the same concentration in aqueous solution, we employed Captisol^®^, a modified cyclodextrin, as a carrier system. As a hydrotropic agent, Captisol^®^ can increase the solubility of flunisolide and myricetin in saline to the concentration we utilized for *in vitro* experimentation (~10 µM). Either compound was mixed in Captisol^®^ solution to enable intranasal administration. To test the cellular uptake of this carrier system, mice were intranasally treated with a fluorescent probe (NHS-rhodamine) embedded in this carrier. Upon inspection of local lung, mediastinal lymph node, and bone marrow (BM) cells, we found that the carrier not only entered lung cells but also trafficked into the draining lymph node and the BM cells (**Supplementary Figure 1**). Fluorescence signal from the carrier was detected in lung leukocytes and non-leukocyte populations, as well as in the lymph node and BM cells, 4 h after intranasal administration. These results indicated that the effects of our training agents could extend beyond the local tissue, as each training molecule was present in the lung, lymph node, and BM.

We next examined how flunisolide or myricetin enhanced host immune responses during a live pathogen infection, to provide a comprehensive view of the immune response. Using the Captisol^®^ carrier system, we trained mice intranasally with flunisolide or myricetin, then infected them intranasally with live *Listeria monocytogenes* (Lm) (**Figure 1C**). Mice were sacrificed for harvest on day 3 post-infection, when Lm pathogen burden reaches its peak (35). To determine differences in pathogen burden, we measured pathogen burden in organs most affected by Lm infection – the lung, spleen, and liver (35). While no significant differences in weight were observed between the control and treated groups (**Supplementary Figure 2**), organs harvested on day 3 post-infection had significantly lower colony-forming units (CFUs) in mice trained with either flunisolide or myricetin compared to control mice (**Figures 1D, E**). Notably, significant reduction in pathogen burden was measured in the lung after training with flunisolide and in both the spleen and lung after training with myricetin. These results indicated that training with flunisolide or myricetin led to cellular or tissue-level changes that translated into lower pathogen burden in the organs most affected by Lm infection. However, there are multiple possible explanations for this reduction in pathogenesis. The compounds could improve immune function in local tissues or induce enhanced infiltration of circulating leukocytes. To better understand how different organs could be protected to different degrees, we examined the BM hematopoietic landscape after training with flunisolide or myricetin.

### 3.2 Agent-specific changes in the bone marrow hematopoietic landscape are observed at baseline without secondary stimulation

A central question of agent-specific training is the contribution of the different forms of innate training. Innate training has been reported to occur both in bone marrow (BM) hematopoietic cells (called ‘central trained immunity’) and in terminally differentiated innate immune cells in peripheral tissues (called ‘peripheral trained immunity’). Central training is characterized by epigenetic reprogramming and metabolic rewiring of BM hematopoietic stem and progenitor cells (HSPCs), which are translated into functional changes in mature innate immune cells (36–39). In contrast, peripheral training is characterized by modifications in circulating cells and tissues that allow faster and stronger responses to a secondary stimulus (19, 24, 40–42). In either training program, cells return to their baseline inactivated state after training but can enhance their response upon a subsequent stimulus through epigenetic memory.

While tracking the destination of intranasally delivered agents, we found that the fluorescently labeled Captisol^®^ carrier was detected in the local lung, lymph node, and BM cells after intranasal administration (**Supplementary Figure 1**). Interestingly, a major shift in fluorescence signal was detected in the BM cells, whereas only a fraction of the lung leukocytes and non-leukocytes showed a fluorescent signal. In addition, our previous report showed agent-specific TF footprints (32). One possible implication of this agent-specific regulation of TFs is alteration of the BM landscape. Central innate training has been shown to occur in the BM by altering HSPCs. Based on these data, we next sought to investigate the effects of flunisolide and myricetin on the BM hematopoietic landscape.

**Figure 2:**
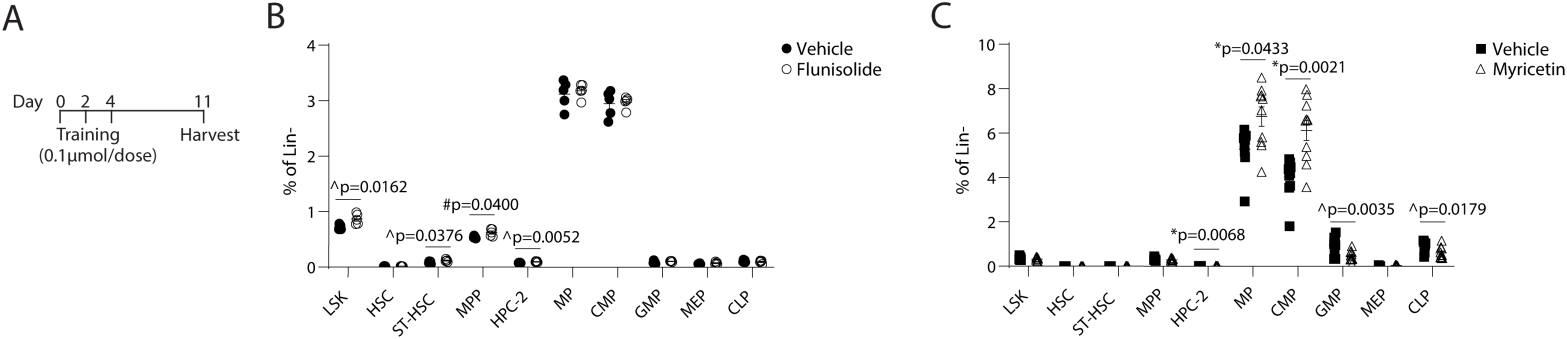
Agent-specific changes occur in the bone marrow hematopoiesis landscape. Flow cytometry analysis of stem cell or progenitor populations as a percentage out of the Lin^-^ population of the isolated bone marrow cells. **(A)** Experimental design of *in vivo* training. Hematopoiesis landscape in the bone marrow is altered towards stem cell and progenitor expansion in flunisolide-trained mice **(B)** or myeloid expansion in myricetin-trained mice **(C)** at baseline. LSK (Lin^-^ Sca-1^+^ cKit^+^ cells), HSC (hematopoietic stem cell), ST-HSC (short-term hematopoietic stem cell), MPP (multipotent progenitor), HPC (hematopoietic progenitor cell), MP (myeloid progenitor), CMP (common myeloid progenitor), GMP (granulocyte-macrophage progenitor), MEP (megakaryocyte-erythroid progenitor), CLP (common lymphoid progenitor). Data is represented as mean ± SEM. *Mann-Whitney test, #Welch’s t-test, ^Unpaired t-test.

To investigate this, we trained mice with flunisolide or myricetin with the same schedule as the infection study; but instead of infecting the mice, we harvested the BM one week after the last training dose, without a secondary stimulus (**Figure 2A**). The purpose of this experiment was to determine whether training induced baseline changes that could subsequently be translated into changes in peripheral tissues. Phenotypic analysis of the BM cells revealed that training with both flunisolide and myricetin induced significant shifts in population composition, despite the absence of secondary stimulation. In flunisolide-trained mice, the hematopoietic stem cell and multipotent progenitor populations were significantly expanded (**Figure 2B**). On average, flunisolide training resulted in 19% greater proportion of LSK (Lin^-^ Sca-1^+^ cKit^+^) population; over 39% of short-term hematopoietic stem cell (ST-HSC) population; over 15% of multipotent progenitor (MPP) population; and, over 24% of hematopoietic progenitor cell-2 (HPC-2) population compared to the control group. No significant changes were observed in oligopotent progenitor cells. In contrast, after training with myricetin, almost all significant changes observed in mice were in oligopotent progenitor cells, namely myeloid lineages (**Figure 2C**). On average, myricetin training resulted in over 50% greater proportion of common myeloid progenitor (CMP) population and over 25% of myeloid progenitor (MP) population compared to the control group. Interestingly, over 30% greater proportion of common lymphoid progenitor (CLP) population was observed on average, further suggesting a bias towards myeloid lineages after myricetin training. These results indicate that myricetin expands more specialized progenitor cells in the BM, while flunisolide expands less restricted stem cells (**Figures 2B,C**).

Together, these results show that intranasal doses of flunisolide and myricetin each induced baseline changes in the BM population. However, these changes were distinct for each molecule, corroborating the initial hypothesis that distinct epigenetic changes result in distinct changes in cellular populations. Given the differences in cell populations and their distinct responses to an infectious challenge, we hypothesized that baseline shifts in the BM population would alter cell infiltration into the site of infection.

### 3.3 Key myeloid functions and cell subsets are enhanced after training with flunisolide or myricetin

**Figure 3:**
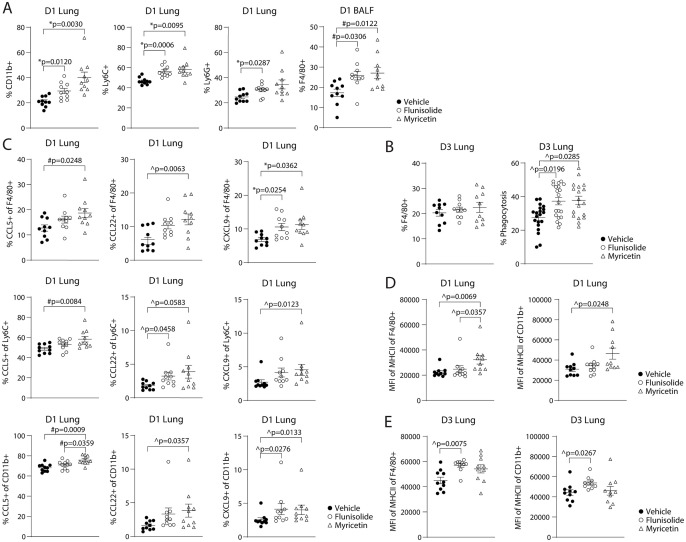
Multiple myeloid cell functions are altered by training. Flow cytometry analysis of leukocyte cell subsets, phagocytosis efficiency, intracellular chemokine production, and surface expression level of MHCII of cells isolated from lungs of mice infected with *Listeria monocytogenes*. **(A)** Training with flunisolide or myricetin leads to a significantly higher percentage of myeloid cells at the site of infection on day 1 post-infection with *L. monocytogenes*. **(B)** F4/80+ macrophages from flunisolide- or myricetin-trained mice on day 3 post-infection exhibit significantly higher phagocytosis of pHrodo beads *ex vivo*. **(C)** Training with flunisolide or myricetin leads to a significantly higher percentage of myeloid cells producing T cell chemokines on day 1 post-infection. Myeloid cells of myricetin-trained mice express significantly higher surface levels of MHCII on day 1 post-infection **(D)** while those of flunisolide-trained mice express significantly higher surface levels of MHCII on day 3 post-infection **(E)**. Data is represented as mean ± SEM. *Welch’s ANOVA with Dunnett’s correction, #Ordinary ANOVA with Tukey’s correction, ^Kruskal-Wallis test with Dunn’s correction.

To determine whether the skewed BM landscape after training alters cell recruitment after infection, we surveyed the lung and bronchoalveolar lavage fluid (BALF) of infected mice at multiple time points after Lm infection. We wanted to understand how differences in the BM hematopoietic landscape might lead to distinct immune responses. In both training modalities, significant increases in proportions of myeloid cell populations at the site of infection were observed by day 1 post-infection (**Figure 3A**). Significantly greater percentages of CD11b+, Ly6C+, or F4/80+ cells were present in the lung at day 1 post-infection in trained mice compared to controls. On average, flunisolide training led to over 39% greater proportion of CD11b+ cells; over 20% of Ly6C+ cells; and, over 49% of F4/80+ cells compared to the control group. Training with myricetin, on average, led to over 90% greater proportion of CD11b+ cells; over 24% of Ly6C+ cells; and, over 56% of F4/80+ cells compared to the control group. In addition, significantly greater proportion of Ly6G+ cells was present in flunisolide-trained mice compared to the control, with an average greater proportion of over 22%, but not in myricetin-trained mice. Interestingly, this trend of Ly6G+ populations is corroborated by our observations in the BM. Myricetin training, on average, led to over 50% lower proportion of granulocyte-macrophage progenitor (GMP) population compared to the control (**Figure 2C**). No such trend was observed in flunisolide-trained mice. Furthermore, training with myricetin resulted in the greatest presence of myeloid cell populations in both the lung and BALF, compared to flunisolide training. As previously shown, a skewed expansion of myeloid lineages after myricetin training was measured in the BM (**Figure 2C**), further supporting our observations after infection. These results indicate faster recruitment of innate immune cells into the site of infection.

In addition to cell recruitment, we wanted to understand how cell function was altered. The recruited myeloid cells after training expressed significantly higher intracellular levels of chemokines, including C-C motif chemokine ligand (CCL)-5, CCL-22, and chemokine ligand (CXCL)-9, in the trained mice compared to the control (**Figure 3C**), revealing that training led to more robust cellular production of chemoattractant cytokines. In addition, training with myricetin or flunisolide led to greater phagocytic efficiency in F4/80+ macrophages (**Figure 3B**). On day 3 post-infection, lungs were harvested and F4/80+ macrophages were isolated using magnetic bead isolation. These cells were then cultured with pHrodo beads *ex vivo* to measure their phagocytosis efficiency. Although the proportion of F4/80+ leukocytes in the lung was not different between the control and treated groups, cells isolated from trained mice showed greater phagocytic efficiency than those from control mice. Together, these data suggest that training with flunisolide or myricetin enables faster recruitment of innate immune cells and enhances their effector functions.

Prior studies show that adaptive immune response to Lm infection is biphasic, with phenotypically and functionally different T cell responses arising early at 24 h post-infection and later by day 7 post-infection (43). Such adaptive response to Lm infection led us to question whether the effects of flunisolide or myricetin on innate leukocytes could affect the ensuing adaptive response. To address this, we first focused on the effects of training on antigen-presenting cells (APCs), as the interaction between APCs and T cells marks the transition from the innate to the adaptive response. We measured the expression level of major histocompatibility complex class II (MHCII) on the surfaces of cells that infiltrated the site of infection at different time points. Interestingly, we observed temporal differences in levels of MHCII surface expression between flunisolide- and myricetin-trained mice. In myricetin-trained mice, significantly higher expression of MHCII was observed in F4/80+ and CD11b+ cells by day 1 post-infection (**Figure 3D**). In contrast, significantly higher expression of MHCII was observed in flunisolide-trained mice on day 3 post-infection, reaching a level higher than that of control or myricetin-trained mice (**Figure 3E**). Notably, the expression level of MHCII continued to increase from day 1 to day 3 post-infection in flunisolide-trained mice, while that of myricetin-trained mice plateaued by day 1 post-infection. These results suggest that an agent-specific effect on innate immune cells leads to significantly different trends in the regulation of MHCII expression. Since we observed a delayed increase in MHCII surface level in flunisolide-trained mice, we decided to inspect the expression of other molecules related to APC function.

### 3.4 Flunisolide induces stronger and faster onset of APC-T cell interaction compared to myricetin

**Figure 4:**
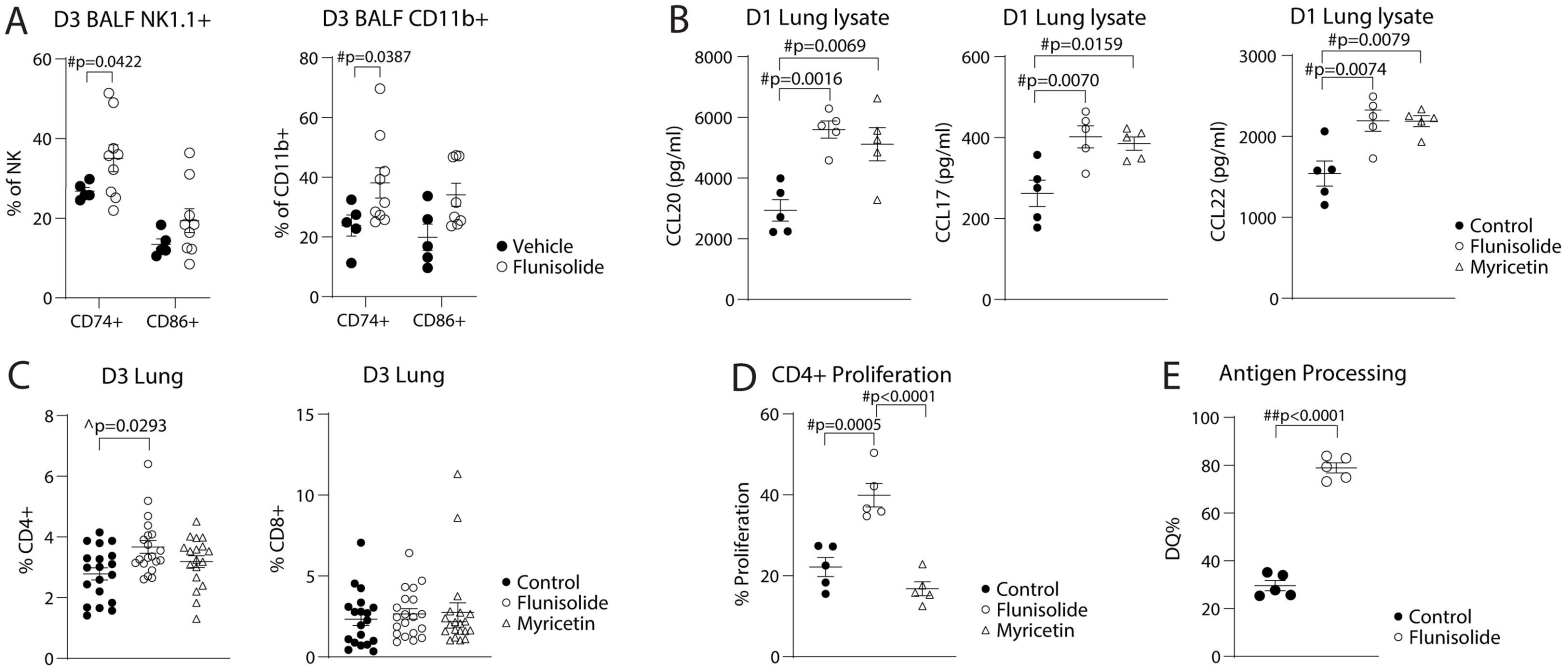
Flunisolide increases APC-T cell interaction and proliferation. **(A)** Flow cytometry analysis of surface receptors on cells isolated from BALF at day 3 post-infection. CD11b+ and NK1.1+ cells express significantly higher levels of CD74 in flunisolide-trained mice. **(B)** Bead-based multiplex assay of chemokines in bulk lung lysate at day 1 post-infection. Flunisolide or myricetin training leads to significantly greater levels of CCL20, CCL17, and CCL22 in the local infected tissue. **(C)** Flow cytometry analysis of CD4+ or CD8+ T cells as a percentage out of CD45+ cells in the lung at day 3 post-infection. Flunisolide-trained mice have a significantly higher percentage of CD4+ T cells at the site of infection on day 3 post-infection. **(D)** *In vitro* expansion assay on bulk splenocyte culture, shown as a % of total cells that underwent proliferation on day 3 post-infection. Flunisolide training led to faster expansion of CD4+ T cells *in vitro*. Data is represented as mean ± SEM. #Ordinary ANOVA with Tukey’s correction, ^Kruskal-Wallis test with Dunn’s correction, ##Unpaired t-test.

Based on our observation of the delayed increase of MHCII in flunisolide-trained mice, we questioned whether flunisolide would affect the resolution phase of the inflammatory response. To answer this, we characterized the expression of CD74 and costimulatory molecule CD86 on innate immune cells at the site of infection. We found that on day 3 post-infection, NK1.1+ and CD11b+ cells in the BALF exhibited significantly higher CD74 expression (**Figure 4A**). On average, we measured over 30% greater proportion of CD74+ surface expression in NK1.1+ cells and over 15% in CD11b+ cells. We also observed a trend towards higher expression of CD86 in CD11b+ cells. We did not observe such differences in myricetin-trained mice (**Supplementary Figure 3**). This indicates that flunisolide induced a distinctive training response that results in a shift toward greater expression of molecules related to APC function.

We previously observed that training led to significantly higher cellular production of chemokines by recruited myeloid cells (**Figure 3C**). To test whether flunisolide or myricetin also affects the local tissue response to Lm infection, we next measured bulk levels of inflammatory cytokines and chemokines. We found that both training agents significantly increased lung levels of T cell-recruiting chemokines (CCL-20, CCL-17, CCL-22) at day 1 post-infection (**Figure 4B**). Collectively, these data suggested that changes in T cell recruitment may be observed in trained mice. To determine whether increased chemokine levels and enhanced APC characteristics altered T cell kinetics, we characterized CD4+ and CD8+ cell populations on day 3 post-infection. Interestingly, we observed a significantly greater proportion of CD4+ population in the infected tissue only in the flunisolide-trained mice; flunisolide-trained mice had over 30% greater proportion of CD4+ cells compared to the control mice. In contrast, no significant differences in the CD8+ population were observed. (**Figure 4C**). Our data on BM hematopoietic landscape after flunisolide or myricetin training corroborate these findings. Training with myricetin led to over 30% lower proportion of CLP population compared to the control, while such difference was not observed after flunisolide training (**Figures 2B,C**). Two possible explanations for the significantly higher proportion of CD4+ cells in the flunisolide-trained mice are that more T cells were recruited due to the high level of T cell-recruiting chemokines or that greater expansion of T cells were induced in flunisolide-trained mice. To understand which scenario was more likely, we harvested spleens from naïve mice and cultured splenocytes *in vitro*. Bulk splenocytes were trained with flunisolide or myricetin, following the same training schedule as *in vivo* (**Figure 1C**). At day 3 post-infection, we observed a significantly higher rate of proliferation in the flunisolide-trained cells compared to the control, but not in the myricetin-trained cells (**Figure 4D**). This marked difference suggests that training with flunisolide alters APC-T cell crosstalk at the later stages of the inflammatory response, an effect not observed with myricetin training.

As flunisolide training led to greater surface expression of MHCII, we next asked whether it would also improve antigen processing. To test this, bulk splenocytes were trained *in vitro*, using the same training schedule as *in vivo* (**Figure 2A**). On day 11, DQ-Ovalbumin (DQ-Ova) was added to the cells and incubated for 4 h. Upon intracellular processing of DQ-Ova, a fluorescence signal is emitted. We discovered that flunisolide training led to 160% more cells that processed the antigen on average compared to the control group, indicating enhanced antigen processing in flunisolide-trained cells (**Figure 4E**). Interestingly, this early increase in proliferation of CD4+ T cells is distinct from the usual adaptive immune response to Lm infection; a previous study showed that two separate populations of regulatory T cells (Tregs) arise after Lm infection that shapes the cytotoxic CD8+ T cell response (43). Collectively, our data support that training with flunisolide, but not myricetin, increases the expression of molecules related to T cell activation in APCs and may facilitate early expansion of CD4+ T cells in the infected tissue. To this point, we have observed key differences between flunisolide and myricetin in their ability to train and then instruct the immune system at distinct points in the response to Lm infection. Training with flunisolide led to increase in antigen processing, elevated MHCII and CD74 expression on APCs, and significantly faster CD4+ T cell expansion – all effects related to the onset of the adaptive response. In contrast, these effects were not observed after training with myricetin; myricetin training led to rapid innate cell recruitment, enhanced phagocytosis, and robust chemokine production – all functions related to the initial innate response. With this in mind, we next questioned what molecular mechanism could be behind these distinct training outcomes.

### 3.5 Differences in downstream regulation of transcription modulate immune response after training

It has been hypothesized that, alongside the alteration of epigenetic signatures, there are changes to the transcriptional program in innate immune memory (17, 44). We have seen differences in how flunisolide and myricetin change both the epigenetic and functional landscape of innate and adaptive responses. These training agents, however, may target different points in the transcriptional pathway. We wanted to test if the differences in epigenetic changes we observed previously and the functional changes we have measured within this paper could be explained by changes in transcriptional activity after training. For flunisolide, the primary molecular target is the glucocorticoid receptor (GR) (45, 46). Upon binding to GRs, flunisolide acts as an agonist, triggering downstream signaling that ultimately blocks the transcription of pro-inflammatory genes. Specifically, the physical association of GRs with nuclear factor (NF)-κB gene sites via tethering or coactivator competition blocks the transcription of downstream pro-inflammatory genes (47, 48). In addition, GRs increase the binding affinity of the inhibitor of κB (IκB) to the NFκB complex, slowing the nuclear translocation and DNA-binding activity of NFκB (49, 50). For myricetin, the primary molecular targets are intracellular kinases, including the mitogen-activated protein kinase kinase (MAP2K) family (51, 52). Myricetin has been reported to act as an inhibitor of these kinases, modulating downstream signaling cascades like the p38 MAPK and the extracellular signal-regulated kinases 1 and 2 (ERK1/2) pathways (53, 54). We sought to test if flunisolide or myricetin differentially modulates the expression and activation of key downstream inflammatory transcription factors.

To test this idea, we treated primary bone marrow-derived macrophages (BMDMs) with flunisolide or myricetin for 24 h *in vitro*, then harvested cell lysates before or after LPS stimulation for 1 h. We used a shortened LPS stimulation period to capture agent-mediated alterations at the transcriptional level. This 24 h priming protocol has been previously utilized in mechanistic studies of trained immunity to directly measure changes incurred by a training agent (34, 36, 55). Although the cells were primed for a shorter time than in our *in vivo* model, differential modulation of molecular pathways by flunisolide or myricetin could indicate the dynamic changes that occurred during a pathogen infection.

To compare the modulation of transcriptional pathways by flunisolide and myricetin, we measured total and phosphorylated protein levels of NF-κB and activating transcription factor (ATF)-2/7. After normalization to β-actin protein levels, we compared transcription factor protein levels between the control and treated groups. Also, we calculated the ratio of phosphorylated to total protein levels of NF-κB or ATF-2/7 to determine how the inhibition of the agent targets was changing the phosphorylation and signaling in the cells. We hypothesized that, given both compounds demonstrated involvement in the regulation of NF-κB and MAPK pathway, there should be alterations to either the level of phosphorylated or total protein consistent with activity.

**Figure 5:**
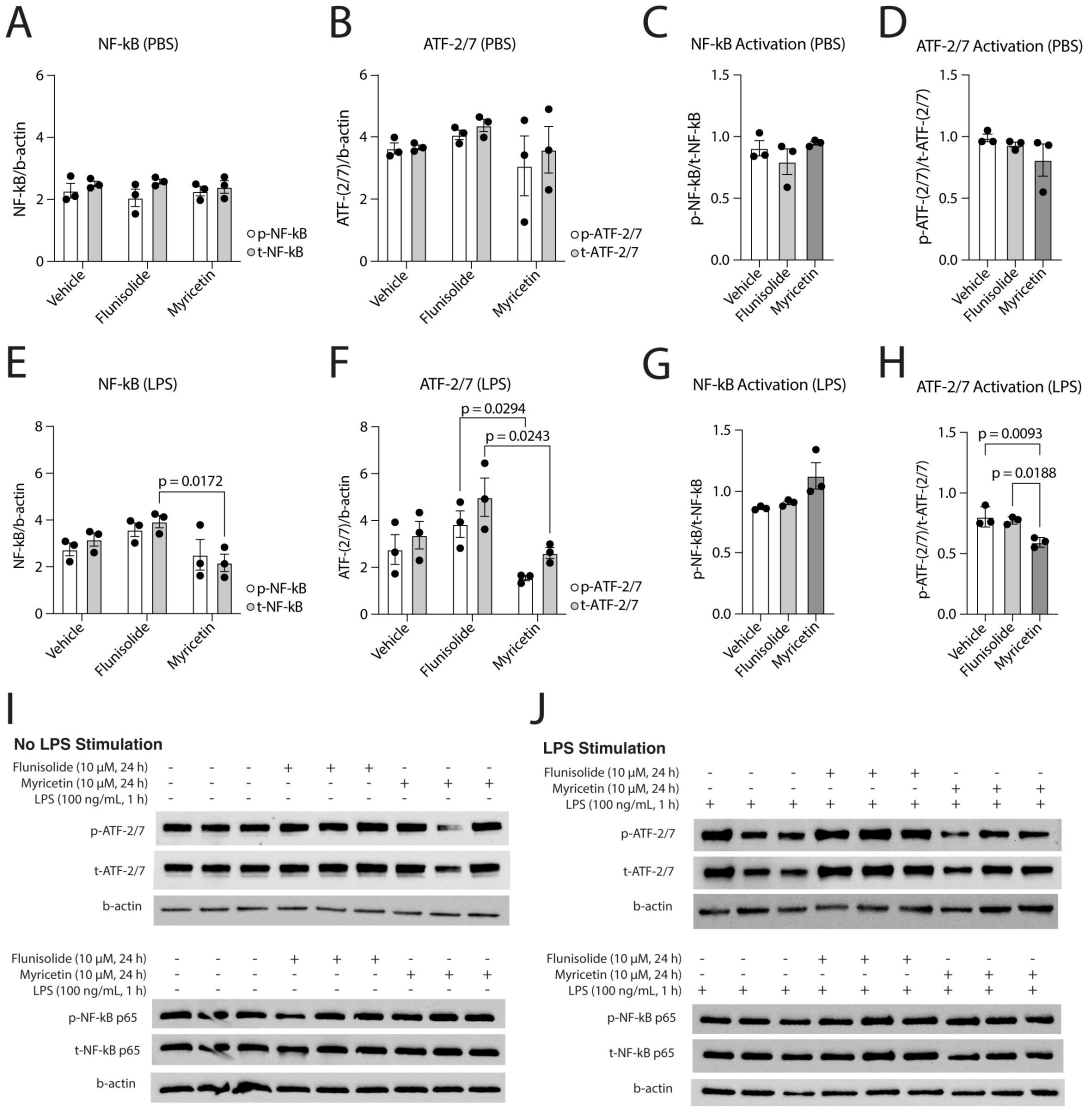
Modulation of NF-κB and ATF-2/7 is agent-specific. Western blot analysis of phosphorylated or total protein level of NF-κB and ATF-2/7 in primary BMDMs treated with flunisolide or myricetin (10 µM) for 24 h and harvested before **(A–D)** or after stimulation with LPS (100 ng/mL) for 1 h **(E–H)**. Total levels of NF-κB, phosphorylated-NF-κB, ATF-2/7, and phosphorylated-ATF-2/7 without **(A, B)** or after 1 h LPS stimulation **(E, F)** in myricetin and flunisolide treated cells are normalized to that of β-actin. Activation levels of NF-κB and ATF-2/7 without **(C, D)** or after 1h LPS stimulation **(G, H)** in myricetin and flunisolide treated cells are represented as the ratio of phosphorylated protein to total protein. Images of immunoblots used for quantification in Panels **A–D (I)** and Panels **E–H (J)**. Data is represented as mean±SEM, One-way ANOVA with Tukey’s correction **(C, D, G, H)**, Two-way ANOVA with Tukey’s correction **(A, B, E, F)**.

After 24 h exposure to the agents, the levels of phosphorylated or total NF-κB or ATF-2/7 (**Figures 5A, B**), nor the activated ratios of NF-κB or ATF-2/7 (**Figures 5C, D**), differ significantly compared to the control. To help elucidate the differences in signaling, we stimulated the cells with LPS. After 1 h, myricetin treatment significantly reduced the ATF-2/7 activation ratio compared with the control and flunisolide-treated groups (**Figure 5H**). No differences were observed in the activated ratio of NF-κB after LPS stimulation (**Figure 5G**). Interestingly, the total protein levels of NF-κB and ATF-2/7, as well as the phosphorylated protein levels of ATF-2/7, were significantly lower in the myricetin-treated cells compared to those of flunisolide-treated cells upon LPS stimulation (**Figures 5E, F**). However, no differences were observed when comparing to the control group. While myricetin treatment led to changes in the levels of NF-κB and ATF-2/7, such changes were not observed after flunisolide treatment. However, our previously published data (32) and the *in vivo* work shown here suggest that both myricetin and flunisolide induce molecular changes that lead to epigenetic and functional differences. NF-κB and ATF-2/7 were not explicitly affected by flunisolide in the 24 h timeframe; however, the results suggest that the differential training programs induced by the two agents are modulated via transcriptional regulation (32).

To further assess whether flunisolide and myricetin alter transcription, we used immortalized BMDMs (iBMDMs) to test the same hypothesis. Immortalized cell lines are reported to have a distinct transcriptional state compared to primary cells due to the genetic and epigenetic modifications incurred to maintain their immortalized state. Due to their uniformity and ease of culture, iBMDMs serve as a useful platform for large-scale studies, such as high-throughput screening. Here, we wanted to test whether treating iBMDMs with flunisolide or myricetin would lead to transcriptional modulation similar to that observed in primary BMDMs. As with primary BMDMs, iBMDMs were treated with flunisolide or myricetin for 24 h, then harvested before or after 1 h LPS stimulation. While no significant changes were observed after LPS stimulation in regard to the levels of phosphorylated or total NF-κB and ATF-2/7 or the activated ratio of NF-κB (**Supplementary Figures 7E, F, G**), we found that flunisolide treatment led to significantly higher activated ratio of ATF-2/7 **(Supplementary Figure 7H**). In contrast to primary BMDMs, iBMDMs showed more pronounced modulation before LPS stimulation. Myricetin treatment led to significantly lower total levels of NF-κB (**Supplementary Figure 7A**), and flunisolide treatment led to significantly higher levels of both phosphorylated and total ATF-2/7 compared to those of the control or the myricetin-treated group (**Supplementary Figure 7B**). Furthermore, the ATF-2/7 activation ratio was significantly higher after flunisolide treatment than in the control (**Supplementary Figure 7D**).

In summary, myricetin training led to decreased levels of NF-κB and ATF-2/7 after LPS stimulation with minimal effects seen after flunisolide training in primary BMDMs. In contrast, training with flunisolide led to significant increases in levels of phosphorylated and total ATF-2/7 before LPS stimulation in iBMDMs. While both are macrophages derived from the bone marrow, primary and immortalized BMDMs exist in different transcriptional states but were both affected by myricetin or flunisolide to differentially express NF-κB and ATF-2/7. Although largely correlative, together, these results show that flunisolide and myricetin may differentially modulate downstream inflammatory transcription factors.

## 4 Discussion

Our work examines how the modulation of distinct molecular pathways can lead to distinct innate immune outcomes. A major question in the field of trained immunity is whether all training molecules operate through a common or distinct mechanism to enhance protection against pathogen challenge. Complex, heterogeneous biologically-derived molecules, such as β-glucan, can limit mechanistic studies because they elicit both training and non-training effects. Small molecules, with their more defined mechanisms of action, ease of formulation, and comparable dosing regimens, provide a direct means to probe how different pathways lead to distinct training outcomes. Here, we examined how two distinct training outcomes by small molecules led to host protection against *Listeria monocytogenes* (Lm) infection. While both agents significantly reduced the Lm pathogen burden, we found that they boosted distinct phases of the inflammatory response. Flunisolide training led to a significant increase in the hematopoietic stem and progenitor cells in the bone marrow (BM). This change in the BM increased protection against Lm challenge by improving antigen processing by APCs and increasing the number of activated T cells. This change may be related to the increased levels of NF-κB and ATF-2/7 upon stimulation after flunisolide training. In contrast, myricetin led to a significant expansion of myeloid lineages in the BM. Training with myricetin led to more rapid and extensive infiltration of innate immune cells into the site of infection and to more robust effector functions, as evidenced by increased chemokine production and phagocytic efficiency. As evidenced by the previously reported epigenetic changes and the changes in transcriptional factors reported here, training with myricetin appears to alter the levels and activity of transcription factors most associated with innate immune function. For flunisolide, changes in activation of transcription factors were associated with a stronger induction of the adaptive immune response over a short time frame, albeit with a slower innate response. Each distinct training program may have different benefits for future applications.

Corticosteroids, like flunisolide, are generally reported to alleviate inflammation (46). Interestingly, we observed an opposite trend, with flunisolide training leading to greater levels of NF-κB and ATF-2/7 upon LPS stimulation (**Figures 5E, F**). In contrast, myricetin training led to lower activated ratio of ATF-2/7 upon LPS stimulation (**Figure 5H**). This balancing of ATF-2/7 expression and activation may explain why myricetin avoids hyperinflammation during training while boosting the overall immune response. These results may suggest the distinctive effects of flunisolide and myricetin on the regulation of transcription factors. Further studies on the kinetics of transcription factor regulation could shed light on the molecular mechanisms underlying flunisolide- and myricetin-induced training. Furthermore, perturbation studies using GR antagonists or knock-out mouse models could help validate the mechanistic roles of transcription factors behind flunisolide- and myricetin-induced training.

With this in mind, we speculate on how agents traditionally considered anti-inflammatory can elicit pro-inflammatory responses with a delayed effect. We have confirmed here and in previous work that these compounds exhibit anti-inflammatory effects within a 24 h window (32). These results may appear to be in contrast with our results at first sight, but the timeline of activity is the key differentiator that helps explain the results. Alterations in inflammatory signaling over 24 h result in epigenomic changes that persist and alter the response upon rechallenge. We report here that exposure to either of these agents led to a more robust innate cell response, as reflected in cell recruitment, chemokine production, and effector function after Lm infection, all of which are part of the pro-inflammatory response. However, recent studies have suggested that the NF-κB signaling pathway is dynamically and combinatorially regulated (56). In addition, previous study shows that coactivation of GRs and NF-kB alters their signaling pathways and the repertoire of regulated genes (57). These data suggest that the effects of flunisolide and myricetin reported in this study manifest as an indirect, opposing effect on the balance of this intricate signaling pathway. By inhibiting one component of a signaling pathway, the effects of another kinase may become more robust. Such balancing of kinase signaling has been previously reported. For example, mitogen-activated protein kinase kinase 2 (MEK2) has been shown to be essential for the MKK3/MKK6-p38 MAPK axis but not for the downstream ERK1/2 pathway (58). While we cannot draw a definitive conclusion for our current observations, the evidence suggests that the alteration of the signaling process is one of the earliest and most easily measured changes in the cell’s state associated with differences in the trained immunity response.

Previous studies have reported that trained APCs enhance T cell responses by increasing expression of MHCII and CD80/86 and by elevating production of inflammatory cytokines. Studies have shown that trained monocytes enhance antigen presentation and T cell polarization (59, 60). Others have reported on the essential role of T cells in macrophage memory formation (28, 61). These results all point to the close interaction between APCs and T cells that enables innate immune cell training. Classical definition of trained immunity is confined to the enhancement of innate immune cell functions, such as cytokine production, and does not include its effects on the adaptive immune system. The effects of flunisolide on innate immune cells, however, were shown to extend beyond this classical definition in this study. In this study, we report increased MHCII and CD86 expression in cells following flunisolide training. In addition, we observed a significant increase in CD4+ T cell proliferation in flunisolide-trained splenocytes. In contrast, we did not observe any changes in CD8+ T cell populations after flunisolide training. While cytotoxic CD8+ T cells are the primary immune cell responsible for clearing Lm infection, it has been shown that CD4+ T cells undergo rapid expansion 24 h post-Lm infection which is critical for the subsequent CD8+ T cell responses and expansion, which occur 5–7 days post-Lm infection (43). We observed significantly greater CD4+ T cell population (**Figure 4C**) and lower pathogen burden (**Figure 1D**) at day 3 p.i., an earlier timepoint than when CD8+ T cells have been shown to expand in response to Lm infection. Together, these data suggest that flunisolide training enhances APC function and T cell responses that contribute to lowering pathogen burden, a unique effect not previously reported. The differences in innate and adaptive responses between myricetin and flunisolide indicates that distinct, agent-specific responses can lead to different methods of protection. Notably, slow but persistent antigen presentation, coupled with enhanced T cell activation, can lead to rapid T cell expansion in the local environment after challenge.

One area that remains to be addressed is the response of the local non-immune, structural cells to the training agents. Structural cells, such as epithelium, endothelium, or fibroblasts, play a significant role in local immune response (62–64). Previous reports have shown the epigenetic plasticity of epithelial cells resulting in robust local protection against pathogens (65–67). As the vehicle, Captisol^®^, penetrated local non-immune cells, it is possible that flunisolide or myricetin induced training in those cells. However, our results show that chemokine production is largely from infiltrated immune cells (**Figure 3C**). In addition, histological analysis of infected lungs did not reveal differences in epithelial structural integrity after infection between control and trained mice (**Supplementary Figure 6**). Together, our results suggest that the observed immune reactions largely originate from the immune cells, not the non-immune structural cells. An area of further investigation is elucidating which immune cell type is directly affected by the training agents.

In summary, we report here two distinct training programs induced by flunisolide and myricetin that shape distinct phases of the inflammatory response against *Listeria monocytogenes* (Lm) infection. We show that flunisolide and myricetin alter distinct populations within the bone marrow hematopoietic landscape and that these two agents differentially modulate inflammatory transcription factors, thereby enhancing multiple aspects of innate immune function. These findings provide mechanistic insight into the differential effects of small molecule training modalities and highlight potential avenues for exploring the modulation of inflammatory responses through distinct training programs.

## Data Availability

The original contributions presented in the study are included in the article/**Supplementary Material**. Further inquiries can be directed to the corresponding author.
