# Supplementary material for: Agent-specific training directs distinctive immune response against Listeria monocytogenes infection
Source: Front Immunol. 2026 Jul 24;17:1868138. doi: 10.3389/fimmu.2026.1868138 (PMC13447383; doi:10.3389/fimmu.2026.1868138)
Supplement: Supplementary file 1 [file Supplementaryfile1.pdf]

Supplementary Material

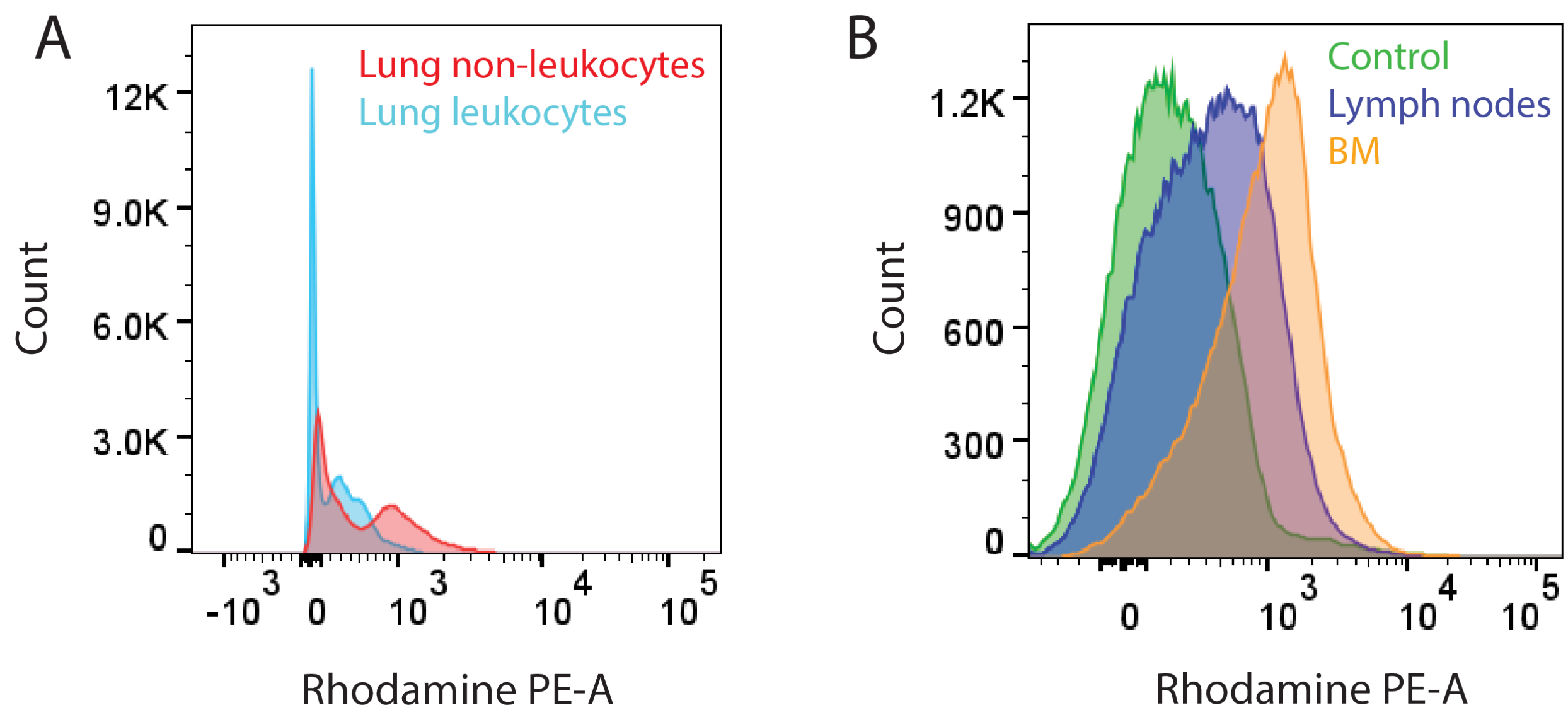

Supplementary Figure 1. Flow cytometry analysis of rhodamine-encapsulated Captisol®. Representative flow cytometry plots of lung **(A)**, mediastinal lymph node, and bone marrow cells **(B)** at 4 h after intranasal administration of rhodamine-encapsulated Captisol®. Shifts in rhodamine signal can be observed in comparison to the unstained control.

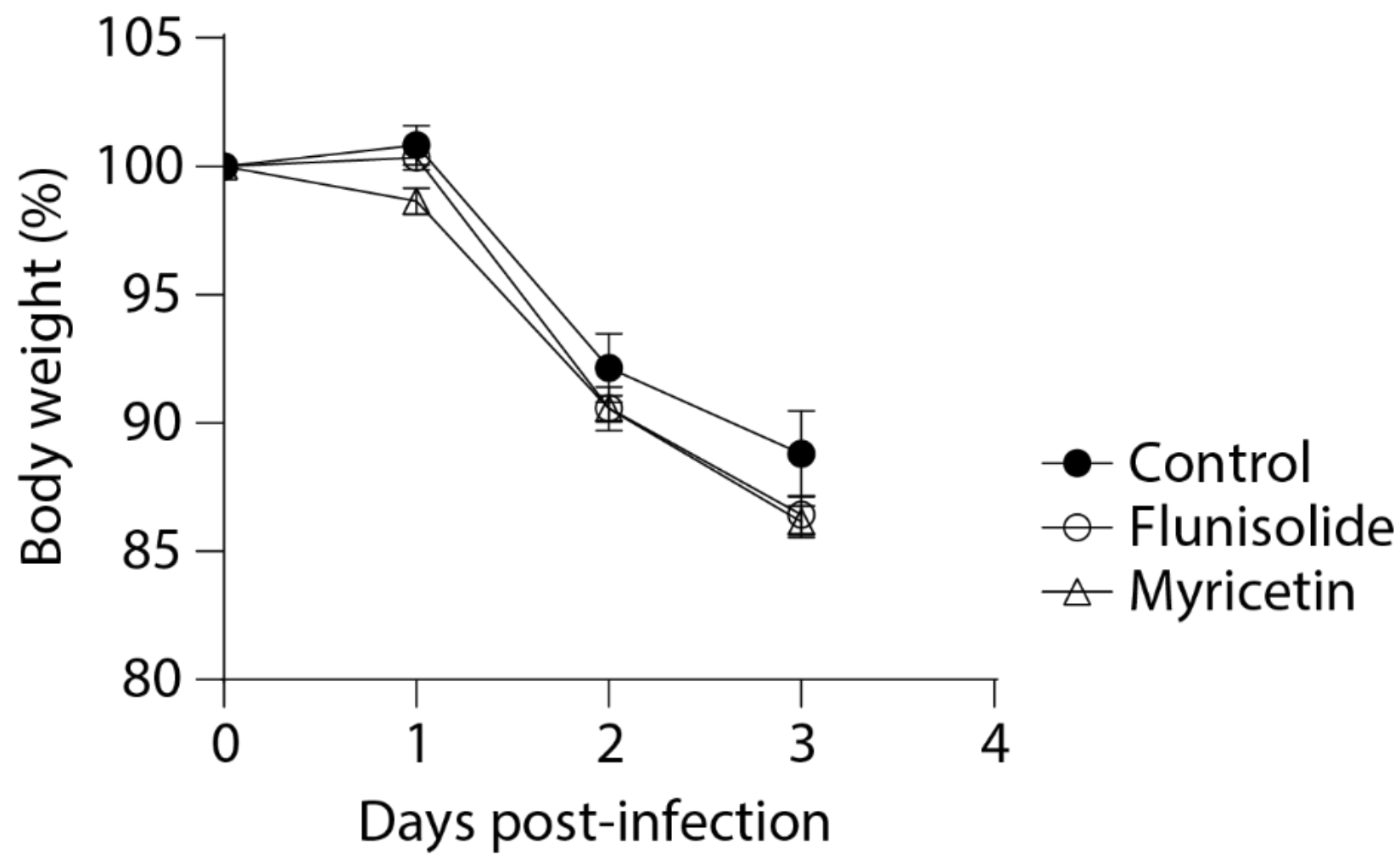

Supplementary Figure 2. Body weight changes post-infection with *Listeria monocytogenes*. No significant differences in body weight were seen between control and treated groups. Data is represented as mean±SEM.

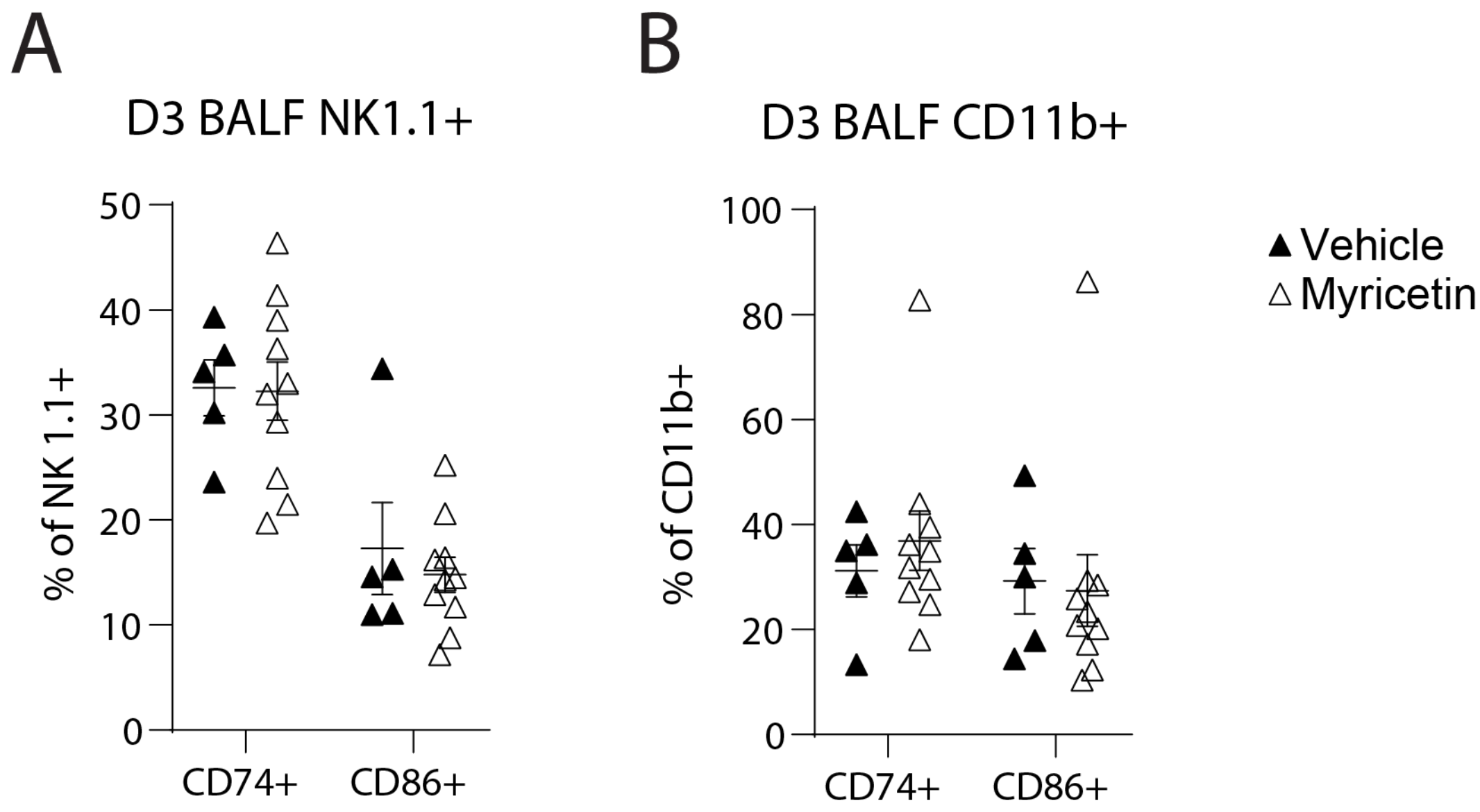

Supplementary Figure 3. Flow cytometry analysis of surface receptors on cells isolated from BALF at day 3 post-infection. NK1.1+ **(A)** and CD11b+ **(B)** cells do not express significantly higher levels of CD74 or CD86 in myricetin-trained mice. Data is represented as mean±SEM.

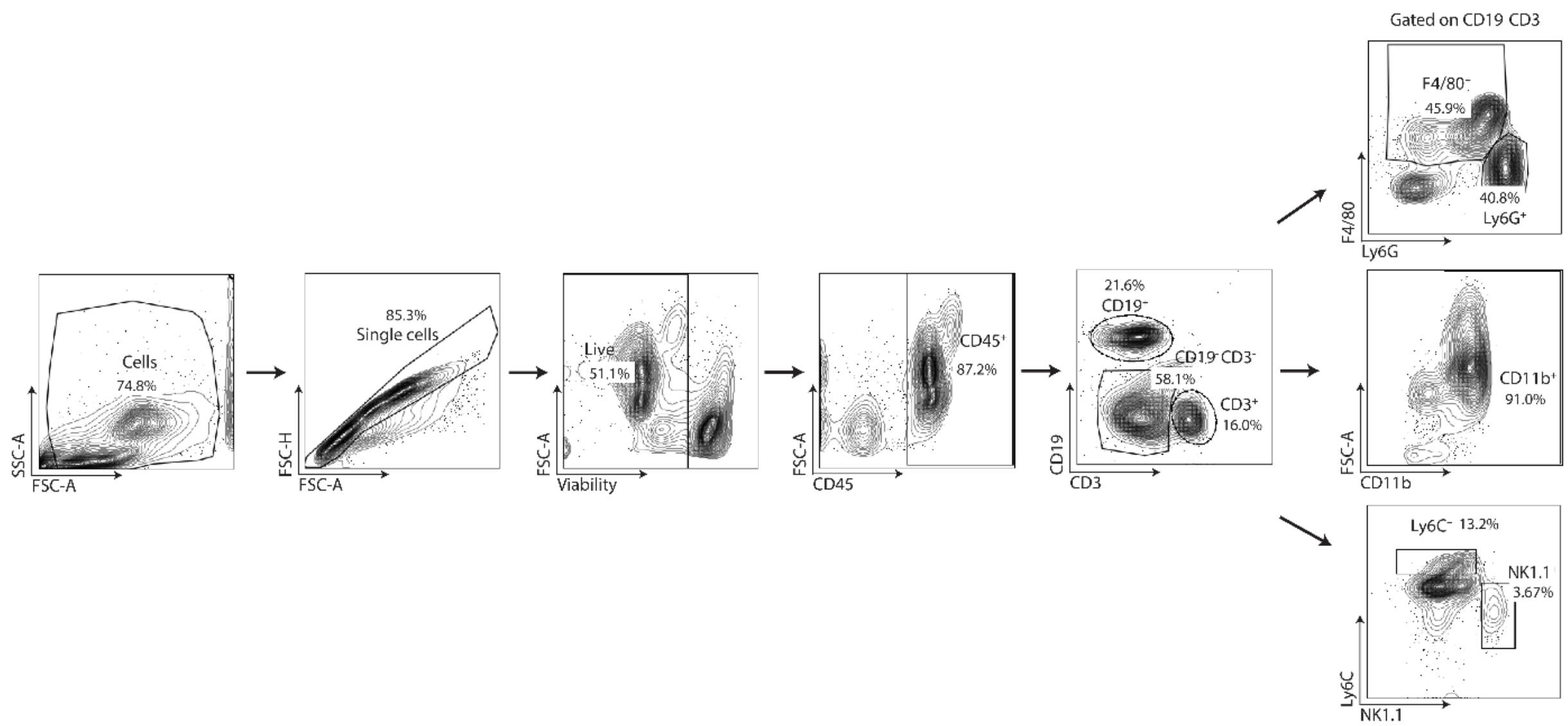

Supplementary Figure 4. Gating strategy to identify leukocytes in lung and bronchoalveolar fluid (BALF). Representative flow cytometry plots to identify leukocyte subtypes in the lung or BALF of mice.

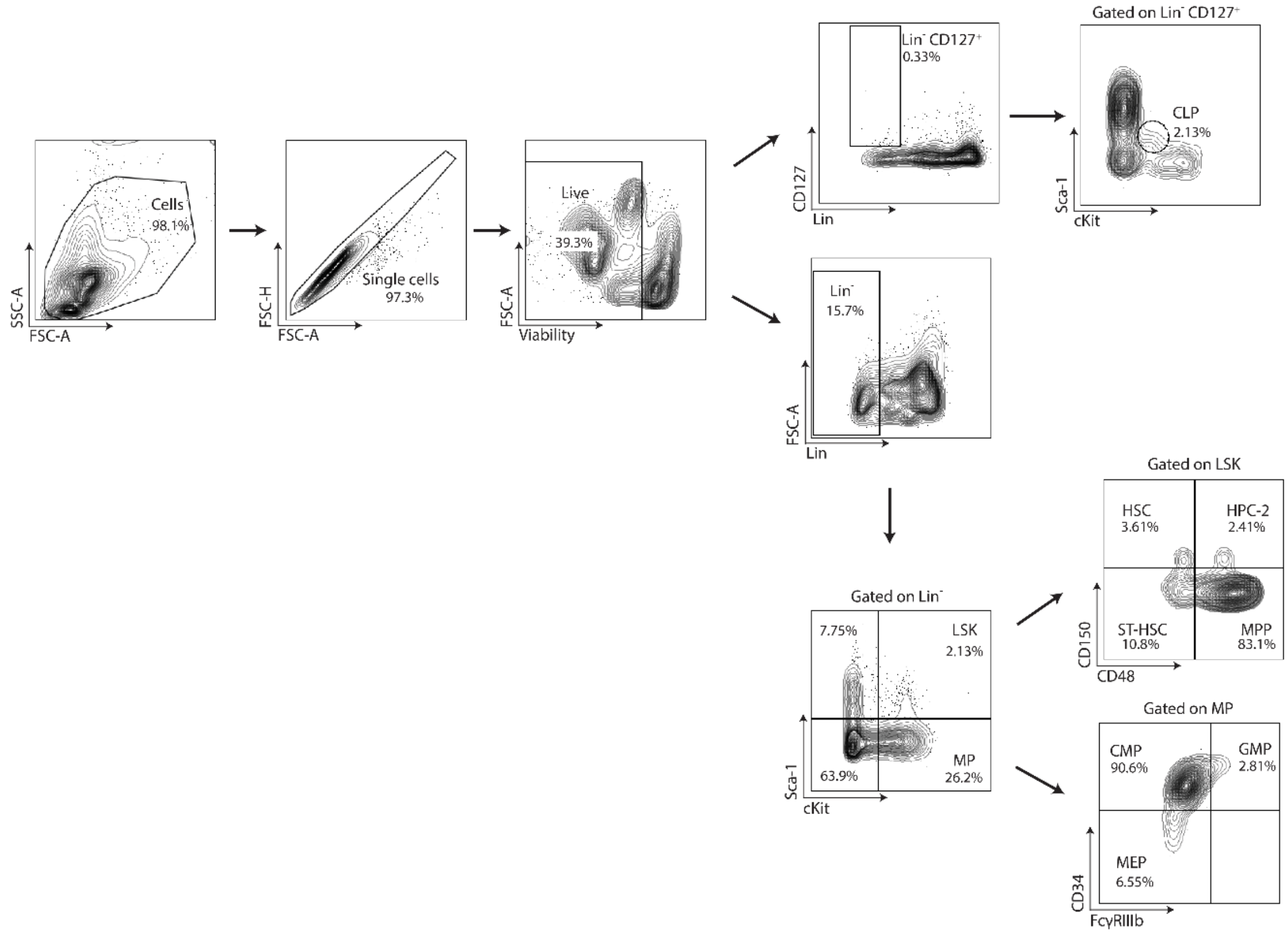

Supplementary Figure 5. Gating strategy to identify subpopulations of hematopoietic stem and progenitor cells in the bone marrow. Representative flow cytometry plots of HSC, HPC-2, ST-HSC, MPP, CMP, GMP, MEP, and CLP cells in the bone marrow of mice.

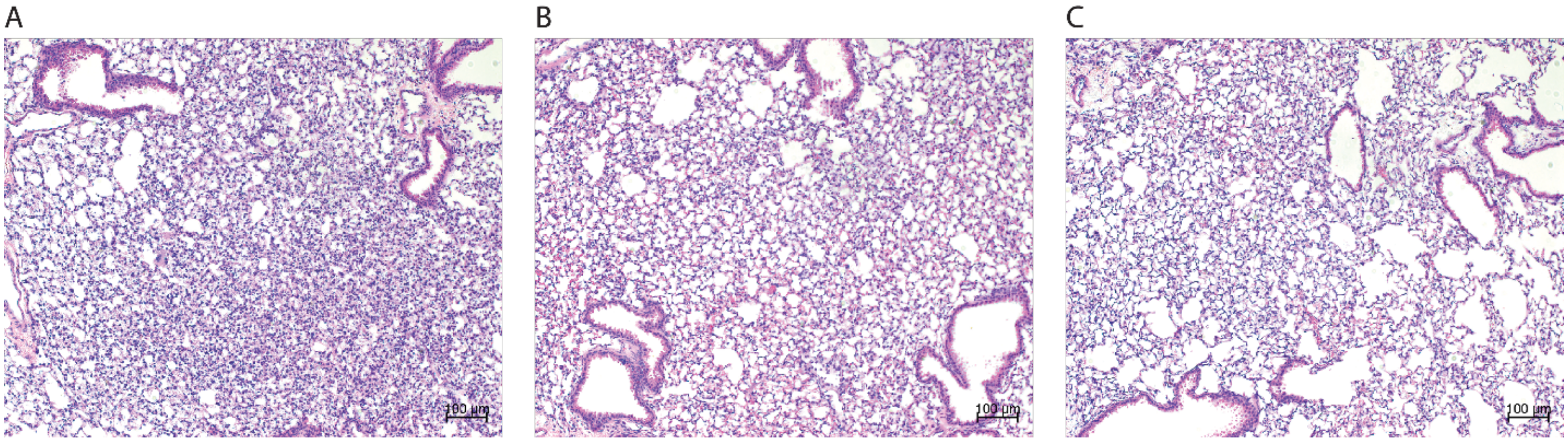

Supplementary Figure 6. Representative histology images of infected lungs. Lungs were harvested 3 d post-infection from control **(A)**, flunisolide-trained **(B)**, and myricetin-trained **(C)** mice and stained with Hematoxylin and Eosin (H&E).

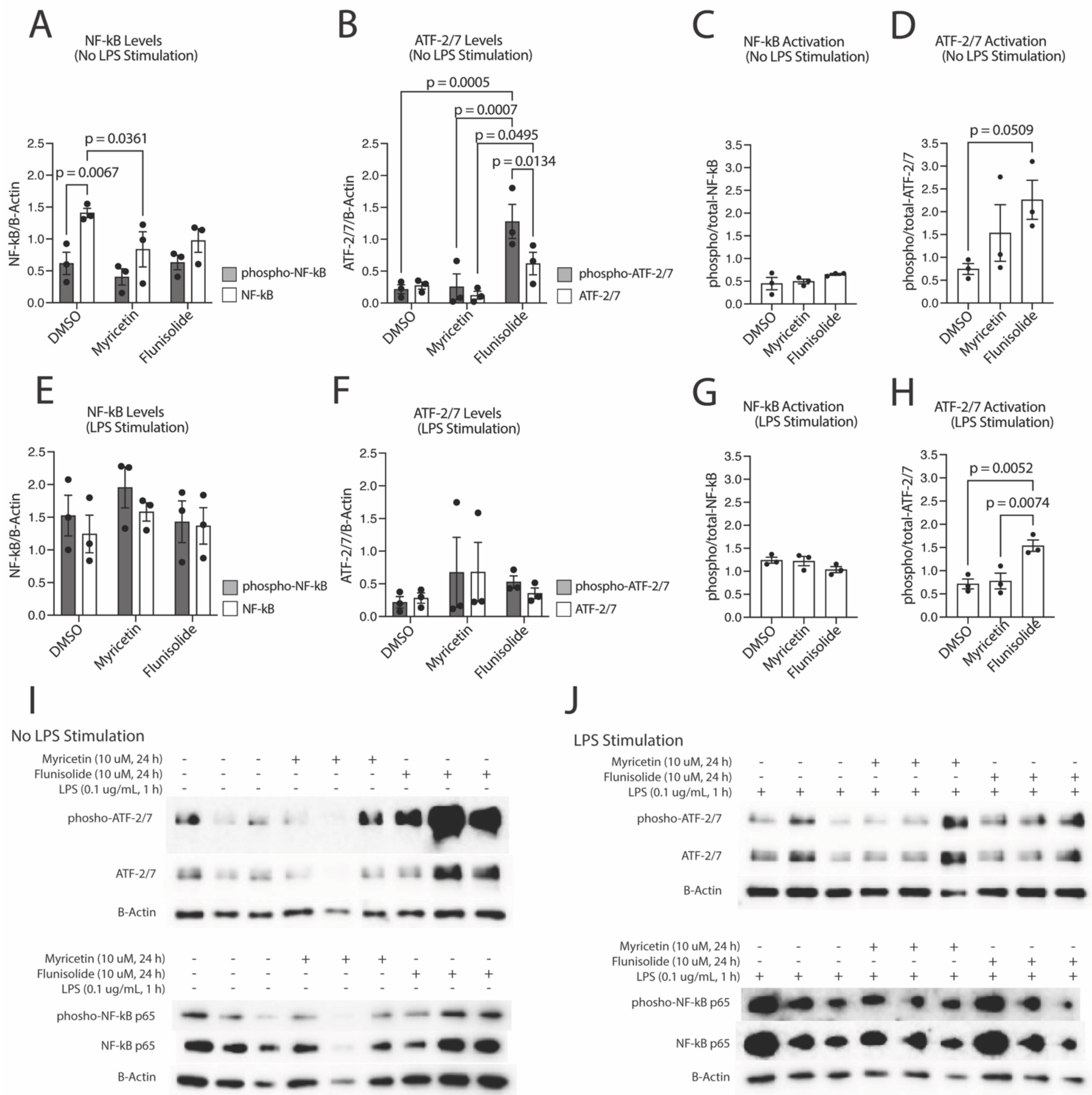

Supplementary Figure 7. Western blot analysis of phosphorylated or total protein level of NF-κB and ATF-2/7 in immortalized BMDMs treated with flunisolide or myricetin (10 µM) for 24 h. Cells were harvested before **(A-D)** or after stimulation with LPS (100 ng/mL) for 1 h **(E-H)**. Total levels of NF-κB, phosphorylated-NF-κB, ATF-2/7, and phosphorylated-ATF-2/7 without **(A,B)** or after 1 h LPS stimulation **(E,F)** in myricetin and flunisolide treated cells are normalized to that of β-actin. Activation levels of NF-κB and ATF-2/7 without **(C,D)** or after 1h LPS stimulation **(G,H)** in myricetin and flunisolide treated cells are represented as the ratio of phosphorylated protein to total protein. Data is represented as mean±SEM, One-way ANOVA with Tukey's correction **(C,D,G,H)**, Two-way ANOVA with Tukey's correction **(A,B,E,F)**.
